# Response to comment on “Alert, but not alarmed” – a comment on “Towards more accurate HIV testing in sub-Saharan Africa: a multi-site evaluation of HIV RDTs and risk factors for false positives (Kosack et al. 2017)”

**DOI:** 10.7448/IAS.20.1.22098

**Published:** 2017-06-19

**Authors:** Cara S Kosack, Anne-Laure Page, Greet Beelaert, Tumwesigye Benson, Aboubacar Savane, Anne Ng’ang’a, Bita Andre, Jean-Paul BN Zahinda, Leslie Shanks, Katrien Fransen

**Affiliations:** ^a^ Médecins Sans Frontières, Amsterdam, The Netherlands; ^b^ Epicentre, Paris, France; ^c^ Institute of Tropical Medicine, Antwerp, Belgium; ^d^ Ministry of Health Uganda, Kampala, Uganda; ^e^ Laboratoire National de Reference, Conakry, Guinea; ^f^ National AIDS and Sexually Transmitted Infections Control Programme, Nairobi, Kenya; ^g^ Regional Delegation of Public Health for the Littoral Region, Yaoundé, Cameroon; ^h^ Programme National de Lutte contre le Sida et les IST (PNLS), Bukavu, Democratic Republic of Congo

Dear editors,

We thank the respondents for their interest, and are glad that they agree overall with our findings and conclusions. We would like to emphasize that we reported [1] on the diagnostic accuracy of individual HIV rapid diagnostic tests (RDTs) and risk factors for false-positive RDTs rather than on the performance of an entire HIV diagnostic algorithm. However, poor-performing individual RDTs will logically influence the performance of the algorithm and a combination of tests with poor specificity increases the likelihood of a false-positive diagnosis. Médecins Sans Frontières has experienced this issue in multiple locations [2–4]. The use of WHO-recommended testing strategies [5] instead of a tiebreaker strategy reduces but does not eliminate the risk of misdiagnosis, especially if the algorithm is not properly validated.

The respondents note that “if all study sites used the data from table 2 and adhered to WHO recommendations, all settings could construct a highly accurate testing algorithm”. Highly accurate in this context would refer to a positive predictive value ≥99% [5]. We agree that good RDT-based algorithms can be found for each site, but risk of misdiagnosis remains if algorithms are not properly validated. To construct a highly accurate algorithm, the individual test performances in the target population must be known and the false reactivity of individual samples with multiple tests considered. These factors remain unknown when algorithms rely solely on WHO prequalification data. We model our algorithms using the results of test evaluation (publication in process).

Regarding the query around whether the specimens were characterized correctly and whether this can be assessed from the information in our article, the characterization of specimens was done at the National HIV Reference Laboratory at the Institute of Tropical Medicine, Antwerp, Belgium, using the algorithm described in Figure 1. Notably, the same laboratory was used in evaluations of the WHO prequalification program [6]. Some differences between our evaluation and that of the WHO prequalification remain: we collected samples prospectively versus WHO use of stored, well-characterized samples; the reference standard differed slightly (e.g. we used only one enzyme-linked immunosorbent as screening assay); our samples originated only from sub-Saharan Africa; and we did not attempt to evaluate performance for HIV-2 or during seroconversion, but had an overall larger sample size. Further comparison is hampered by lack of information on the constitution of the WHO HIV reference panel [6].

In addition, our objective was not to reproduce WHO evaluations but to assess diagnostic accuracy of HIV RDT with specimens from different origins and to compare these results with WHO recommendations for designing HIV testing algorithms. The 2015 consolidated guidelines state that the testing strategies for diagnosis described have been developed assuming that all HIV serological assays used should have a sensitivity of >99% and specificity of >98% (lower bounds of the confidence interval) [5], which is why we used this indication as a benchmark in our discussion. Our prospective design allowed for the calculation of these confidence intervals relevant to our study populations but they were often too wide to conclude positively even for tests with good point estimates. Furthermore, our point estimates were often not similar to the ones found in WHO prequalification evaluation. Updated recommendations from the WHO on how to evaluate and validate the accuracy of HIV RDTs and algorithms would be of great benefit to people working in this field [7].

We agree that self-reported malaria was a significant factor for false reactivity, as determined in the multivariate analysis. However, since no further laboratory characterization of the samples for co-infection was made, these results can only hint towards an association that certainly merits further investigation.
